# Spatial distribution and urban–rural disparity of unmet need for family planning among married/in-union women in Ethiopia: a spatial and decomposition analysis

**DOI:** 10.3389/frph.2024.1416280

**Published:** 2024-12-02

**Authors:** Shimels Derso Kebede, Daniel Niguse Mamo, Jibril Bashir Adem, Agmasie Damtew Walle, Yawkal Tsega, Elsabeth Addisu, Zinabu Bekele Tadese, Ermias Bekele Enyew

**Affiliations:** ^1^ Department of Health Informatics, School of Public Health, College of Medicine and Health Science, Wollo University, Dessie, Ethiopia; ^2^ Department of Health Informatics, College of Medicine and Health Sciences, Arba Minch University, Arba Minch, Ethiopia; ^3^ Department of Public Health, College of Medicine and Health Science, Arsi University, Asella, Ethiopia; ^4^ Department of Health Informatics, School of Public Health, Asrat Woldeyes Health Science Campus, Debre Berhan University, Debre Birhan, Ethiopia; ^5^ Department of Health System and Management, School of Public Health, College of Medicine and Health Sciences, Wollo University, Dessie, Ethiopia; ^6^ Department of Reproductive and Family Health, School of Public Health, College of Medicine and Health Sciences, Wollo University, Dessie, Ethiopia; ^7^ Department of Health Informatics, School of Public Health, College of Medicine and Health Science, Samara University, Samara, Ethiopia

**Keywords:** unmet need, family planning, spatial analysis, multivariate decomposition, urban–rural disparity, Performance Monitoring for Action, PMA, Ethiopia

## Abstract

**Background:**

High unmet need for family planning combined with other factors, such as high early marriage and teenage pregnancy, increases the risk of developing obstetric fistula and other complications. This study aimed to assess spatial distribution and urban–rural disparities of unmet need for family planning among married/in-union women in Ethiopia.

**Methods:**

The study was conducted on secondary data from a cross-sectional survey that was conducted nationally between September and December 2019 using a two-stage cluster design on a total of 265 enumeration areas. A total weighted sample of 5,349 married/in-union women was included in the analysis. ArcGIS Pro and SaTScan software were used to handle spatial analysis. Finally, multivariable decomposition analysis via a logit model was used to decompose the observed difference in unmet need by the compositional difference and the difference in effects of explanatory variables between places of residence.

**Results:**

Spatial distribution of unmet need for family planning was clustered in Ethiopia with a global Moran's I index value of 0.25 (*p*-value = 0.004). Accordingly, enumeration areas in West Hararge, Arsi, Bale, Gujji, Borena, Jimma, and East Wellega zones of Oromia region, and Gurage, Hadiya, Silte, Gedio, Sidama, Wolaita, Alaba, and Dawro zones of South Nation and Nationality People region, and the southern part of Zone 3 in Afar region were detected as hotspot areas. The decomposition results revealed that there is a significant disparity in unmet need between urban and rural resident women (0.074, *p*-value < 0.001). Endowment and coefficient factors accounted for the urban–rural disparity, contributing 68.32% and 31.68%, respectively. Household size, husband’s opinion of family planning, community acceptance of family planning, woman’s age at first sexual intercourse, and the woman's age were key determinants of the urban–rural disparity.

**Conclusion:**

The results revealed a significant disparity in the unmet need for family planning based on place of residence, with a clustered spatial distribution across the study area and notable hotspot areas. Thus, targeted interventions should focus on mobilizing resources to high-risk areas and addressing the needs of high-risk groups to reduce the observed variation.

## Background

Defining unmet need is complex and generally refers to the percentage of reproductive-age married/in-union women who wish to delay or avoid pregnancy but are not using any form of contraception (1). This measure reflects the success of reproductive health programs in meeting women’s contraceptive needs and serves as a criterion for evaluating how well a country’s health system responds to women's preferences to delay or avoid childbearing (2). Although there may be many reasons why women do not use contraception, or have an unmet need for family planning (FP), the existence of unmet need often highlights the lack of access to contraceptive services (3).

Although contraceptive use prevents unsafe abortions, pregnancy-related deaths, and unwanted pregnancies, which have serious consequences for the health and wellbeing of women and families, particularly in developing countries (4), unmet need for family planning remains a significant challenge within the health system. A high unmet need for family planning, combined with other factors such as early marriage and teenage pregnancy, increases the risk of obstetric fistula (5). Hence, reducing the unmet need for family planning plays a substantial role in eliminating fistula.

Globally, more than 10% of women experience an unmet need for family planning, with approximately 20% of women in Africa facing this challenge. In 2017, the proportion of family planning demand satisfied by modern contraceptive methods among married or in-union women of reproductive age was lowest in Africa at 56%, compared with more than 75% in all other regions (1). In Ethiopia, the Ministry of Health aimed to reduce unmet need from 24% in 2015 to 10% by 2020; however, the unmet need remains considerably high at 22%, reflecting only a 2% reduction over 5 years (6). Studies also showed unmet need disparities among married women of reproductive age in different regions of Ethiopia, ranging from 6.9% in Addis Ababa to 31.0% in Oromia region (7).

Previous literature indicates that a range of factors, including women's age, education, wealth status, media exposure, age at first sex, household size, healthcare decision-making, knowledge of family planning methods, and husband's disapproval of family planning, are associated with unmet need for family planning (8–10). However, there is limited evidence on the spatial variation of unmet need for family planning, particularly regarding the urban–rural disparity, using recent national representative data and spatial and decomposition analysis. Unmet need for family planning remains a significant barrier to achieving universal reproductive health, a key target of the Sustainable Development Goal on gender equality. Addressing this unmet need, especially given the disparities between urban and rural areas, is essential for reducing unintended pregnancies, improving maternal health, and empowering women's reproductive choices. In alignment with the Sustainable Development Goals, this research supports efforts to reduce unmet need, thereby advancing health equity and reproductive rights. Investigating the geographical distribution using Geographic Information System (GIS) technology is essential for planning interventions tailored to minimize the burden in high-risk areas. Furthermore, understanding the factors contributing to urban–rural gaps will help policymakers recognize the extent of these disparities, enabling the design of more effective interventions. Thus, this study aimed to assess the spatial distribution and the urban–rural disparities of unmet need for family planning among married/in-union women by analyzing data from the recent 2019 Performance Monitoring for Action (PMA) survey. The PMA survey captures changes seen within a year for different levels of interventions as it is collected every year, compared with the Ethiopian Demographic and Health Survey (EDHS), which releases data every 5 years.

## Methods

### Study design and period

The study used secondary data from the 2019 PMA Ethiopia survey, a cross-sectional household and female survey. The survey was conducted between September and December 2019 using a two-stage cluster design, with urban–rural and major regions serving as strata, encompassing a total of 265 enumeration areas (11). The PMA survey collects annual cross-sectional data from women aged 15–49 years across all regions of Ethiopia. The survey is conducted in collaboration with Addis Ababa University, Johns Hopkins University, and the Ethiopian Federal Ministry of Health (12).

### Study population

All women aged 15–49 years who were married or living with a partner in Ethiopia were included in the study.

### Sample size

The sample size of the analysis was weighted to adjust for non-responses and variations in the probability of selection. It was restricted to a total weighted sample of 5,349 reproductive-age women who were married or living with a partner during the survey.

### Study variables

#### Dependent variable

Unmet need for family planning was the dependent variable and was dichotomized into two categories: “unmet need” and “no unmet need.” According to the definition standardized in DHS reports and updated in 2012, unmet need for family planning refers to a fertile, sexually active woman who wants to delay or avoid pregnancy but is not using contraception (13).

#### Independent variables

The independent variables of unmet need for family planning included in the analysis were as follows: woman's age, level of education, wealth status, media exposure, age at first sex, partner’s opinion about FP, knowledge of available contraceptive methods, whether the partner/husband told her not to use FP, household size, and community acceptance of family planning.

### Data processing and analysis

The data were processed using Stata version 17 and multilevel decomposition on logistic regression was fitted. Further spatial analysis was carried out using ArcGIS Pro 2.8 and SaTScan™ 10.0.2.

### Spatial analysis

#### Spatial autocorrelation analysis

Spatial autocorrelation, assessed using Global Moran's I statistic, was applied to assess whether the unmet need for family planning was clustered, dispersed, or randomly distributed in Ethiopia. A Moran's I value close to −1 suggests disease dispersion, while values close to +1 indicate clustering and random distribution if Moran's I value is 0 (14, 15). A statistically significant Moran's I (*p* < 0.05) would indicate the presence of spatial autocorrelation, rejecting the null hypothesis, which suggests a random pattern of unmet need for family planning across the country.

#### Getis-ord Gi* hot spot analysis

Getis-ord Gi* statistics could identify hot spot areas by computing *z*-score to confirm the statistical significance of clustering at *p*-value <0.05 with a 95% confidence interval (16–18). If the z-score is between −1.96 and +1.96, the *p*-value would be >0.05 and the null hypothesis could not be rejected; thus, the pattern could be due to chance whereas if the z-score falls outside the range, the observed spatial pattern is significant, and the *p*-value would be small; thus, the null hypothesis will be rejected (14). Statistical output with a high Gi* value suggests a hotspot, indicating a high proportion of unmet need for family planning in the area, whereas a low Gi* value indicates a coldspot, indicating a low proportion of unmet need for family planning.

#### Spatial scan statistics

In this study, a pure spatial scan statistical analysis was conducted using SaTScan version 10.0 software to identify statistically significant spatial clusters of unmet need for family planning among reproductive-age women. The spatial scan analysis (SaTScan analysis) strengthened the findings from the Getis-Ord analysis, which is powerful in detecting hot spot areas, confirming the results (17). The Bernoulli model was applied, with women experiencing unmet need for family planning considered as cases and those without unmet need as controls. Potential clusters of unmet need were ranked based on their log-likelihood ratio (LLR) test, corresponding *p*-values, and relative risk. Clusters with a *p*-value <0.05 were considered significant hotpot areas, with the primary cluster being the one with the highest LLR, and the remaining clusters categorized as secondary. Finally, the results from the SaTScan analysis were mapped across the study areas to highlight the hotspot areas of unmet need for family planning.

#### Spatial interpolation

Spatial interpolation using a kriging tool was applied to predict unmet need for family planning at in areas that were not originally sampled during the survey.

### Decomposition analysis

A decomposition analysis using a logit model and the *Mvdcmp* package in Stata (19) was applied to understand the factors contributing to the observed disparity in unmet need for family planning between place of residence (i.e., differences in characteristics) and the difference in effects of explanatory variables (i.e., differences in the coefficients) between urban and rural residents. To compare urban and rural respondents in the context of unmet family planning needs, this analysis works by estimating separate logistic regression models for unmet need among urban and rural women, allowing each characteristic's contribution to be assessed individually. It calculates what proportion of the disparity is due to the characteristics themselves, such as education or wealth, and what proportion is due to differences in how these characteristics affect unmet need across urban and rural settings. This approach provides insight into whether the disparity in unmet need is driven more by sociodemographic differences between urban and rural women or by the varying influence of these factors across settings. By decomposing these effects, it identifies which factors contribute most to the observed unmet need disparity. A coefficient with a 95% confidence interval and a *p*-value of 0.05 was used to test the statistical significance of estimates.

To calculate the appropriate confidence interval and other estimates, the Stata software needs to account for the sample design at the start of the decomposition model fitting by using the *svyset* command, which requires the primary sampling unit variable, weight variable, and stratum variable. These variables are “EA_ID,” “FQweight,” and “strata,” respectively, in the PMA survey.

## Result

### Characteristics of participants

A total weighted sample of 5,349 married/in-union women was included in the study, with three-quarters (73.3%) of women being rural residents. The prevalence of unmet need was 14.4% in urban women and 21.9% in rural women. In both urban and rural areas, almost all women had knowledge of any available contraceptive methods. Two-thirds (62.1%) of women in urban areas and nearly one-quarter (28.1%) of women in rural areas had media exposure (i.e., heard about FP on media). Regarding the educational level of respondents, more than half (58.4%) of rural women had no formal education and only 7.5% had secondary and above education. In contrast, only one-fifth (19.1%) of urban women had no formal education and nearly half (45.5%) had attended secondary school and above. More than half (59.5%) of urban women and 36.7% of rural women had fewer than five family members in the household. Economically, more than half (57.1%) of rural women and only 3.8% of urban women were living in poor households. In both urban and rural settings, the majority of respondents were aged 26–35 years, with 43.6% of urban and 38.7% of rural women falling into this age group. More than two-thirds (67.6%) of women in rural settings and less than half (43.1%) of urban women started sexual intercourse before the age of 18 years. Regarding community acceptance of FP, approximately half (49.2%) of urban women and more than half (58.0%) of rural women reported that the community did not accept FP use (Table 1).

**Table 1:** Characteristics of study participants by place of residence among married or in-union women aged 15–49 years in Ethiopia, PMA 2019.

Variable	Category	Place of residence	
		Urban	Rural
Unmet need status	No unmet need	1,225 (85.6%)	3,059 (78.1%)
	Unmet need	206 (14.4%)	859 (21.9%)
Know any contraceptive method	No	4 (0.3%)	60 (1.5%)
	Yes	1,427 (99.7%)	3,858 (98.5%)
Education level	No education	273 (19.1%)	2,290 (58.4%)
	Primary	507 (35.5%)	1,336 (34.1%)
	Secondary and above	650 (45.5%)	293 (7.5%)
Household size	Less than 5	851 (59.5%)	1,438 (36.7%)
	5–10	564 (39.4%)	2,365 (60.4%)
	More than 10	16 (1.1%)	115 (2.9%)
Husband/partner feeling on FP use	Disapprove use	250 (17.5%)	1,060 (27.2%)
	Does not care	150 (10.5%)	645 (16.5%)
	Approve use	1,033 (72.1%)	2,196 (56.3%)
Husband/partner told not to use FP	No	1,152 (81.0%)	3,251 (83.4%)
	Yes	271 (19.0%)	645 (16.6%)
Wealth status	Poor	54 (3.8%)	2,238 (57.1%)
	Middle	60 (4.2%)	994 (25.4%)
	Rich	1,317 (92.0%)	686 (17.5%)
Community acceptance to use FP	Not acceptable	703 (49.2%)	2,266 (58.0%)
	Neutral	56 (3.9%)	150 (3.8%)
	Acceptable	671 (46.9%)	1,492 (38.2%)
Age at first sexual intercourse	Before age of 18	616 (43.1%)	2,643 (67.6%)
	After age of 18	815 (57.0%)	1,266 (32.4%)
Women's age	15–25	515 (36.0%)	1,160 (29.6%)
	26–35	624 (43.6%)	1,517 (38.7%)
	36–49	291 (20.4%)	1,242 (31.7%)
Total		1,431 (26.8%)	3,918 (73.3%)

### Spatial analysis result

#### Spatial autocorrelation

**Figure 1:**
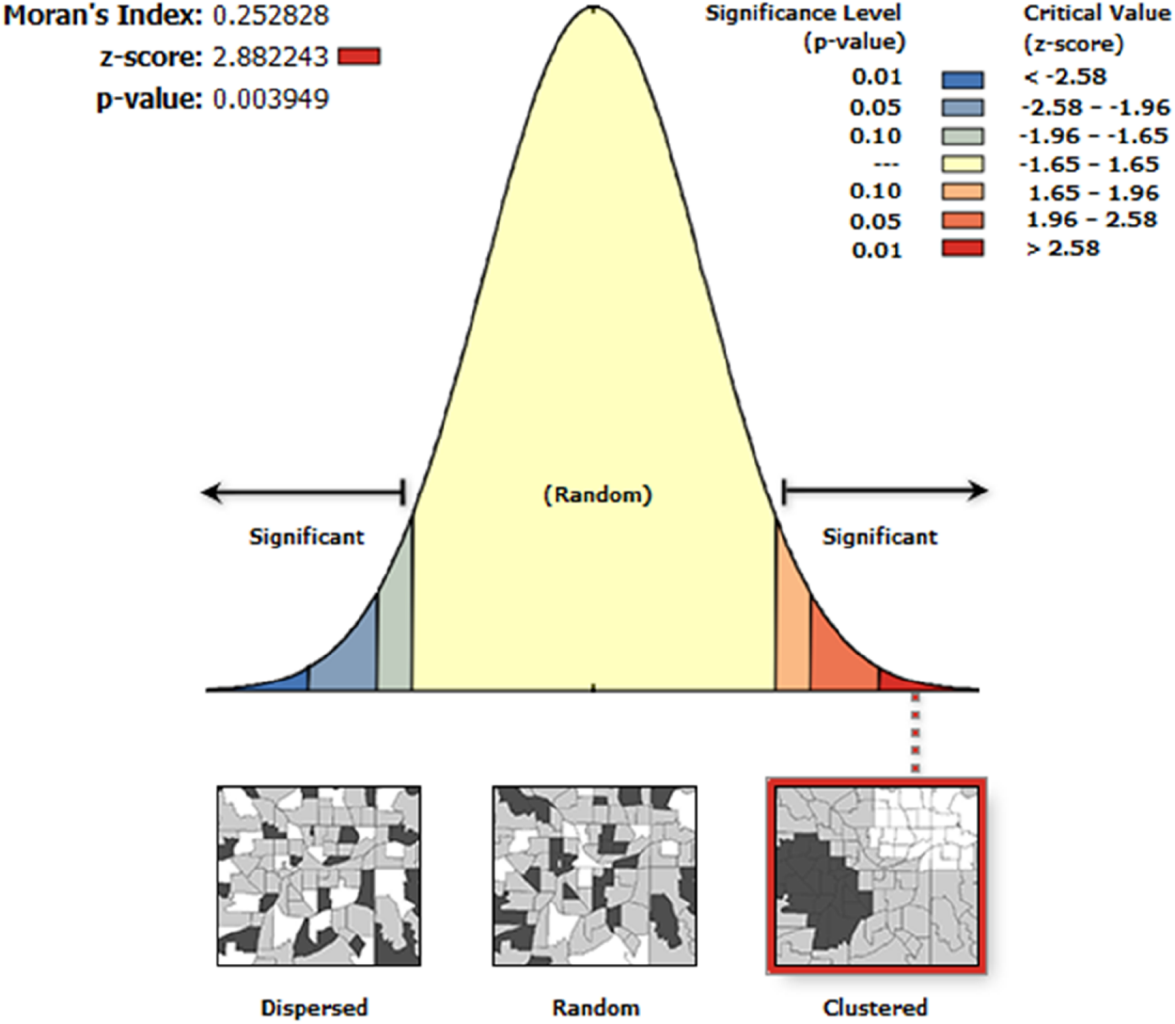
Spatial autocorrelation of unmet need for family planning in Ethiopia, PMA 2019.

The spatial distribution of unmet need for family planning was clustered in Ethiopia. The spatial autocorrelation global Moran's I index value was 0.25 and *Z*-score 2.88 (*p*-value = 0.004), indicating a statistically significant clustering effect (Figure 1). Given the *z*-score of 2.88, there is less than a 1% likelihood that this clustered pattern is due to random chance.

#### Hotspot (Getis-ord Gi*) analysis

**Figure 2:**
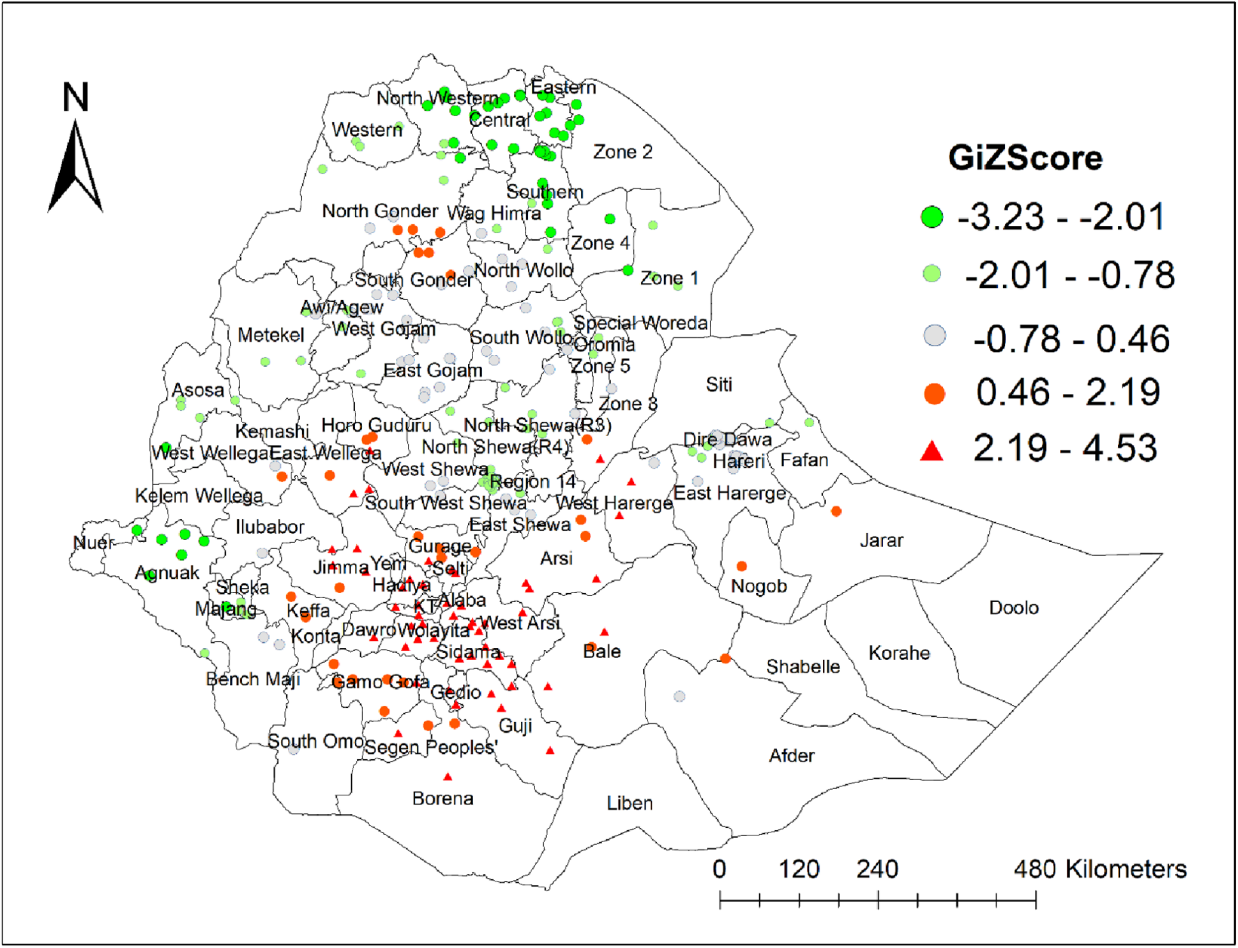
Hotspots and coldspots of unmet need for family planning in Ethiopia, PMA 2019.

The highest risky areas for unmet need for family planning were detected in West Hararge, Arsi, Bale, Gujji, Borena, Jimma, and East Wellega zones of the Oromia region, and Gurage, Hadiya, Silte, Gedio, Sidama, Wolaita, Alaba, and Dawro zones of South Nation and Nationality People (SNNP) region, and the southern part of Zone 3 in Afar region. North and South Gondar zones of Amhara region; West Wollega; and Horo Guduru zones of Oromia region; Keffa and Gamo Gofa zones of SNNP; and Nogob, Jarar, and northwestern part of Shabelle zones in the Somali region were also hotspot areas for unmet need for family planning. In contrast, all parts of the Tigray region, Zone 1 and 4 in Afar region, Wag Himra, the eastern part of South Wollo, Oromia special zone, and North Shewa zone of Amhara region, North Shewa and West Shewa zones of Oromia region, Metekel and Assosa zones of Beshangul-Gumuz region, Gambela region, Sheka and Majang zone of the SNNP region, Addis Ababa city, and Dire Dawa city administrations were coldspot areas for unmet need for family planning (Figure 2).

#### Spatial scan analysis

**Figure 3:**
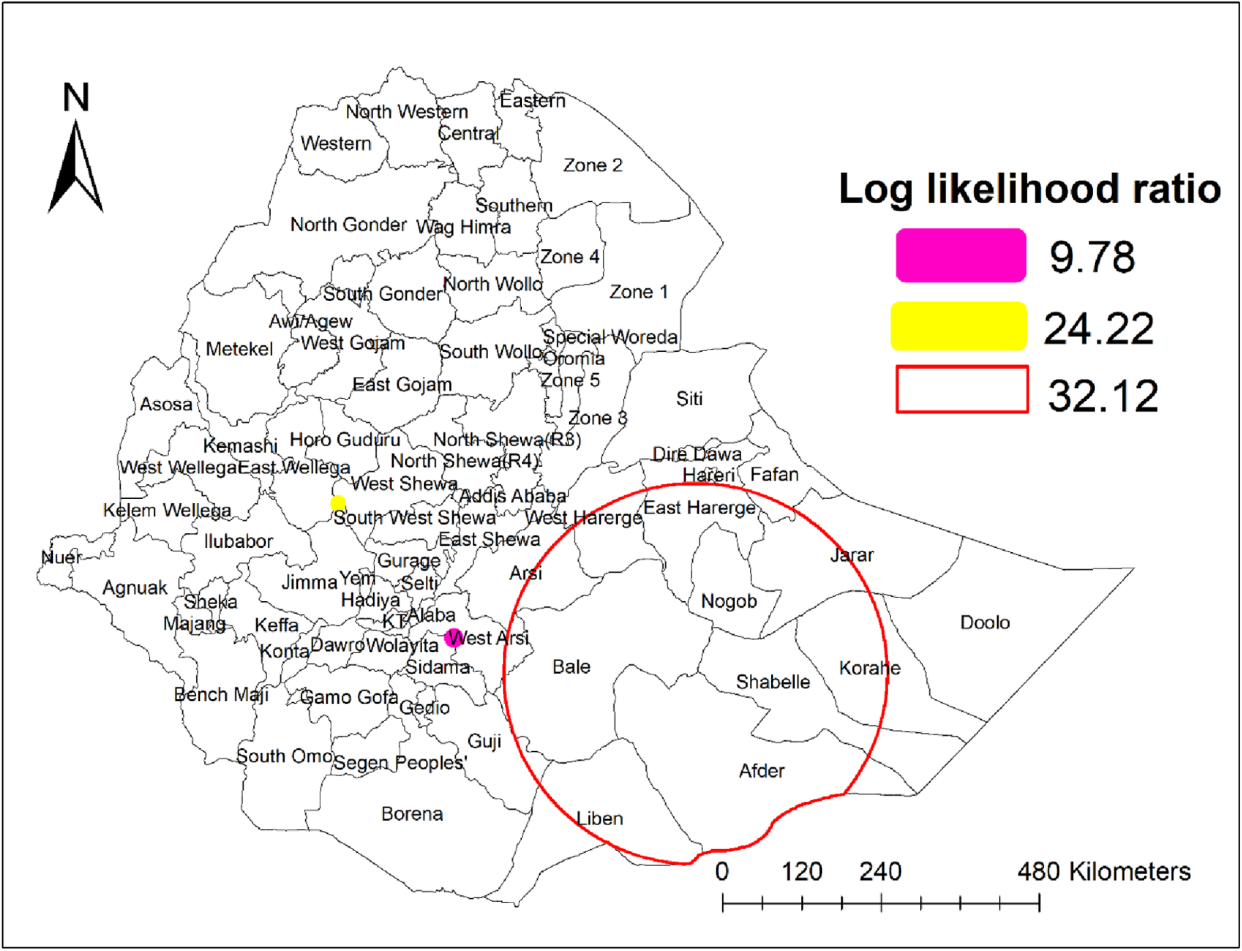
Spatial scan statistics analysis of unmet need for family planning in Ethiopia, PMA 2019.

Purely spatial analysis identified three statistically significant clusters of unmet need for family planning through a maximum spatial cluster size of ≤50% of the population at risk. The SaTScan analysis detected one most likely (primary) cluster and two second most likely (secondary) clusters (Table 2, Figure 3).

**Table 2:** Summary of SaTScan results of unmet need for family planning in Ethiopia, PMA 2019.

Detected cluster	Coordinate/radius	Population	Cases	RR	LLR	*p*-value
Primary cluster	(6.570971N, 41.974927E)*/*288.52 km	735	180	1.89	32.12	0.001
Secondary cluster	(7.058930N, 38.683079E)/0 km	70	34	3.56	24.22	0.001
Secondary cluster	(8.893220N, 37.103871E)/0 km	77	26	2.46	9.78	0.02

The primary cluster for unmet need for family planning was centered at 6.570971N, 41.974927E with a 288.52 km radius and has a relative risk of 1.89 (LLR = 32.12, *p*-value = 0.001). This cluster incorporates the western part of Somali, including Nogob, Shabelle, Afder, Liben, and Jarar, and the eastern part of Oromia, including Harerge, Bale, and Arsi. Those women who lived in enumeration areas within this spatial window were 1.89 times more likely to have unmet need for family planning compared to women outside the window.

The second most likely cluster was detected in a single enumeration area around the borders of East Shewa and West Wellega zones of the Oromia region. It was centered at 7.058930N, 38.683079E within a 0 km radius and had a relative risk of 3.56 (LLR = 32.12, *p*-value = 0.001). Women in this cluster were 3.56 times more likely to have unmet need for family planning compared to women outside this spatial window.

Another significant cluster was detected in enumeration areas on the border between Sidama and West Arsi zones in Oromia region, centered at 8.893220N, 37.103871E with a radius of 0 km. This cluster has a relative risk of 2.46 (LLR = 9.78, *p*-value = 0.02), which means the women within this spatial window were 2.46 times more likely to have unmet need for family planning compared with women outside this cluster.

#### Interpolation

**Figure 4:**
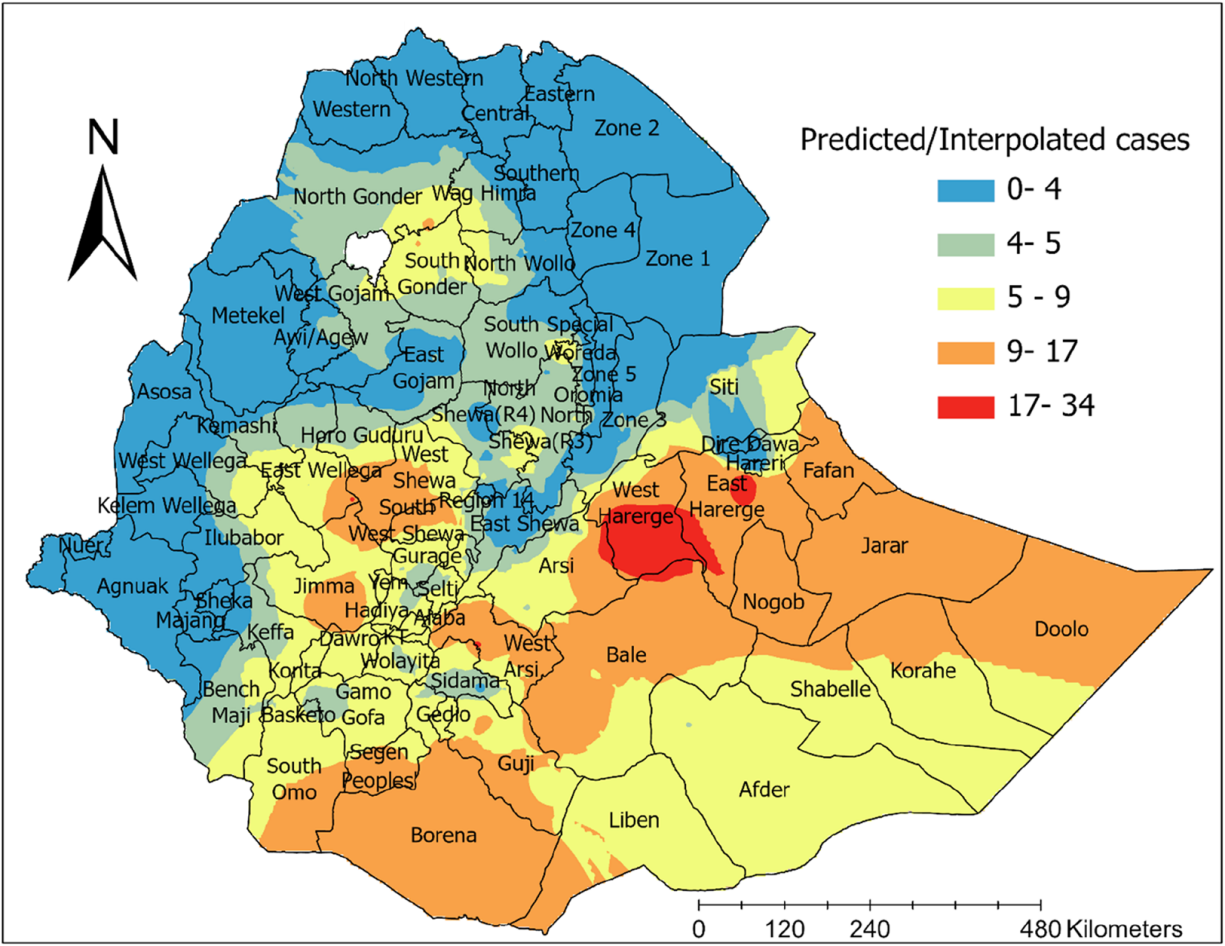
Spatial interpolation of unmet need for family planning in Ethiopia, PMA 2019.

The spatial interpolation result revealed that in areas shaded with dark red color, such as West and Eastern Hararge, the eastern part of Arsi, the northern part of Bale zone, and southeastern part of East Wellega zones in the Oromia region, the highest number of unmet need for family planning was predicted. Furthermore, a high risk of unmet need for family planning was predicted in the northern part of the Somali region and the south and eastern parts of the Oromia region (Figure 4).

### Decomposition result

The detail decomposition result showed that there is a significant disparity in unmet need between urban and rural resident women (0.074, *p*-value < 0.001). The difference in unmet need between urban and resident women was 68.32% due to differences in characteristics and 31.68% due to differences in coefficients effects (difference in behavior). A significant urban–rural disparity in unmet need for family planning was due to household size, husband’s opinion of FP use, community acceptance of FP, age at first sexual intercourse, and women's age.

Controlling for other factors, household size would reduce the urban–rural unmet need disparity by 31% for a family of 5–10 members and by 7% for a household of more than 10 members. That is, if rural women had a household size of 5–10 and more than 10 like urban women, the urban–rural disparity would decrease by 31% and 7%, respectively (*p*-value < 0.001). Community acceptance of family planning would reduce the urban–rural unmet need disparity by 4% (*p*-value = 0.04) if rural women accepted family planning use at the same rate as the urban community. Husband’s opinion of family planning use would decrease the urban–rural unmet need disparity by 10% (*p*-value = 0.02) if rural women’s husbands approved the use of family planning methods at the same rate as urban women’s husbands. If rural women started sexual intercourse before the age of 18 years and were not protected from risks to the same degree as urban women, the urban–rural unmet need gap would be expected to increase by 11.3% (*p*-value = 0.04). Similarly, if rural women aged 36–49 years were not protected from risks to the same degree as urban women of the same age, the urban–rural unmet need gap would be expected to increase by 7.9% (*p*-value = 0.02) (Table 3).

**Table 3:** Detail decomposition result of unmet need for family planning by place of residence among reproductive-age married/in-union women in Ethiopia, PMA 2019.

Decomposition	Coefficient with 95% CI		Percent explained		*p*-value		
Endowment (E)	0.05089 (0.01822 to 0.08355)		68.32		0.002		
Coefficients (C)	0.02360 (–0.01653 to 0.06372)		31.68		0.249		
Interaction (R)	0.07449 (0.04949 to 0.09949)		100		0.000		
		Due to difference in characteristics (E)			Due to difference in coefficients (C)		
Variable	Category	Coefficient	Percent	*p*-value	Coefficient	Percent	*p*-value
Media exposure	Not exposed	Reference			Reference		
	Exposed	−0.00643	−8.63	0.294	0.03045	40.89	0.121
Know any contraceptive method	No	Reference			Reference		
	Yes	−0.00109	−1.46	0.074	0.18547	249.01	0.282
Education level	No education	Reference			Reference		
	Primary	0.00040	0.53	0.096	0.00281	3.77	0.844
	Secondary and above	0.02108	28.31	0.124	−0.02487	0.338	−33.39
Household size	Less than 5	Reference			Reference		
	5–10	0.02311	31.03	0.000*	0.02085	28.00	0.167
	More than 10	0.00505	6.78	0.000*	0.00121	1.62	0.278
Husband/partner feeling on FP use	Disapprove use	Reference			Reference		
	Does not care	0.00005	0.07	0.970	−0.00462	−6.20	0.356
	Approve use	0.00746	10.01	0.018*	−0.01085	−14.56	0.698
Husband/partner told not to use FP	No	Reference			Reference		
	Yes	0.00068	0.91	0.218	−0.01126	−15.12	0.113
Wealth status	Poor	Reference			Reference		
	Middle	−0.00682	−9.15	0.102	0.00449	6.03	0.234
	Rich	−0.00682	23.80	0.274	−0.00837	−11.24	0.884
Community acceptance to use FP	Not acceptable	Reference			Reference		
	Neutral	0.00002	0.03	0.386	0.00023	0.31	0.938
	Acceptable	0.00299	4.01	0.043*	0.00286	3.83	0.836
Age at first sexual intercourse	Before age of 18	−0.00841	−11.29	0.042*	−0.02342	−31.45	0.085
	After age of 18	Reference			Reference		
Women's age	15–25	Reference			Reference		
	26–35	0.00055	0.73	0.608	0.00615	8.25	0.685
	36–49	−0.00586	−7.87	0.023*	−0.00348	4.67	0.691

* *p*-value < 0.05.

## Discussion

This study was conducted to determine the spatial distribution of unmet need in Ethiopia using recent 2019 PMA survey data. Accordingly, the spatial distribution of unmet need was clustered across the study area, which was confirmed by spatial autocorrelation analysis. This result is in line with a study assessing spatial distribution of unmet need based on 2016 EDHS, which revealed non-random distribution of unmet need in Ethiopia (20). Accordingly, new hotspots were detected in West Hararge and Borena zones of Oromia region, North and South Gondar zones of Amhara region, Nogob, Jarar, and north western part of Shabelle zones in Somali region, which were insignificant clusters of unmet need before 2016. In contrast, all parts of Gambela region, and Sheka and Majang zones of SNNP region made a significant shift from hotspots in EDHS 2016 to coldspots for unmet need in 2019. This may be due to the efforts carried out in the last 4 years as part of the HSTP I plan to reduce the unmet need in the hotspots. However, efforts must be strengthened in the coldspots to avoid new occurrences of hotspots. This also suggests that policies and strategies should maintain a balance to ensure coldspots remain at low risk while reducing the burden in the hotspots.

Another aim of this research was to assess the urban–rural disparities in unmet need among married/in-union Ethiopian women using the 2019 survey data. The results highlighted that a significant disparity in unmet need between urban and rural resident women, with women in rural settings of Ethiopia having a higher unmet need than those in urban settings. This finding aligns with previous studies in Ethiopia (21, 22), Bangladesh (23), and India (24), which reported significantly higher unmet need for FP in rural women. More than two-thirds of the observed urban–rural unmet need disparity could be attributed to differences in characteristics. This implies that the urban–rural gap in unmet need would be reduced more by changes in resource distribution (endowments) than by working on behavioral changes of rural women.

The findings of this study showed that household size was a significant factor for narrowing the urban–rural disparities of unmet need for family planning. Controlling for other factors, equalizing rural household size to urban levels at 5–10 family members and more than 10 members would reduce the urban–rural unmet need disparity by 31% and 7%, respectively. This result suggests that the large gap could be reduced by limiting the number of household members to a smaller level. This finding is consistent with a study conducted in Nigeria, which reported lower unmet need for contraception in women who wanted smaller family sizes (25). This could be explained by the fact that women who want smaller family sizes may use contraceptives more carefully, despite various challenges.

Community acceptance of family planning would reduce the urban–rural unmet need disparity by 4% if rural women accepted family planning use at the same rate as the urban community. Results from another study also revealed negative sociocultural factors and social disapproval of contraception are high in rural areas, leading to women's contraceptive utilization (26). This may be due to contraceptive-related myths and false rumors, which lead to social misconceptions, as most of the rural women are less educated and have limited media exposure.

Husband’s opinion of family planning would decrease the urban–rural unmet need disparity by 10% if rural women’s husbands approved of family planning at the same rate as urban women’s husbands. This finding aligns with studies conducted in Ethiopia (10, 22, 27) and sub-Saharan Africa (28), which reported higher unmet need for family planning among women whose husbands disapprove of family planning use. This disparity might be due to women in rural areas having lower decisional autonomy, with husbands typically making important family decisions, including those related to family planning.

Findings from this study also highlighted the influence of age at first sexual intercourse on women's unmet need for family planning. If rural women who began sexual intercourse before the age of 18 years were not protected from risks to the same degree as urban women, the urban–rural unmet need gap would be expected to increase. This might be due to the majority of rural women starting sexual intercourse at an early age, and they may feel ashamed to access family planning services due to community norms in rural Ethiopia, which disapprove of early sexual initiation. In addition, women at this age are often dependent and less aware regarding their reproductive issues.

The urban–rural disparity in unmet need was also influenced by women's age. If rural women aged 36–49 years were not protected from risks to the same degree as urban women of the same age, the gap would be expected to increase. This finding aligns with studies in Ethiopia (29) and Indonesia (30), which reported a positive association between older age and unmet need for family planning. This may be because women approaching menopause often perceive themselves as being at low risk of pregnancy even though they are still capable of childbearing.

While this study presents valuable insights into the spatial distribution and urban–rural disparities of unmet need for family planning in Ethiopia, it is important to acknowledge certain limitations that could inform future research directions. First, it relies on secondary data from the 2019 Performance Monitoring and Accountability Survey, which may not capture all nuances of unmet need for family planning in Ethiopia, particularly in rapidly changing sociocultural contexts. In addition, the cross-sectional design of the study limits the ability to establish causal relationships between the identified factors and the unmet need for family planning, as it reflects a single point in time. Furthermore, self-reported data may be subject to biases, such as social desirability bias, especially in rural settings where discussing family planning may carry stigma.

Despite these limitations, the study also possesses significant strengths. The use of spatial analysis techniques, including ArcGIS Pro and SaTScan, allows for a comprehensive understanding of geographic disparities in unmet need, revealing important hotspots that require targeted interventions. In addition, employing multilevel decomposition analysis provides insights into the factors contributing to urban–rural disparities, enabling policymakers to identify specific areas for intervention.

Drawing from the key findings, this study highlights several actionable policy recommendations. Policymakers should prioritize resource allocation and family planning services in identified hotspot regions to effectively reduce unmet need and improve reproductive health outcomes. Similarly, initiatives to increase community acceptance of family planning methods, particularly in rural areas, should be developed, focusing on dispelling myths and misconceptions while promoting the benefits of family planning through local health campaigns. In addition, strategies to empower women and engage their partners in family planning discussions are crucial. Programs that address husband approval and aim to improve women's decisional autonomy can help close the urban–rural disparity in unmet need.

In light of the findings on the spatial clustering of unmet need for family planning, future research could benefit from a more granular analysis of the sociodemographic factors unique to identified hotspot and coldspot zones. Employing methodologies such as geographically weighted regression (GWR) would enable a localized exploration of determinants, providing insights into how specific community-level characteristics influence unmet need for family planning in these distinct areas. This approach could reveal critical factors that vary across regions and help inform targeted interventions tailored to the unique sociodemographic contexts of hotspot and coldspot areas. By building on the broader spatial insights presented in this study, such localized analyses could further support the development of policies and strategies that address the specific needs of high-risk communities and sustain low unmet need in coldspot areas.

## Conclusion

This study was conducted to determine the spatial distribution and urban–rural disparity of unmet need for family planning in Ethiopia using recent PMA 2019 survey data. Accordingly, the spatial distribution of unmet need for family planning was clustered across the study area. Hotspot areas were detected in Oromia, South Nation and Nationality People, and Afar regions. The spatial interpolation also predicted a higher magnitude of unmet need in Oromia region. The identification and prediction of such high-risk areas provides critical insights for targeted interventions. By focusing on these areas, decision-makers can allocate resources more effectively, ensuring that services reach populations with the greatest need. Tailored interventions, such as community outreach or facility upgrades, can address the specific barriers to family planning in these regions. Moreover, hotspot identification allows for better monitoring and evaluation of interventions, ensuring that strategies are adjusted for maximum impact. Overall, focusing efforts on high-risk areas is essential for reducing unmet need and improving access to family planning services.

The decomposition results revealed a significant disparity in unmet need for family planning between urban and rural women in Ethiopia, with rural women experiencing a higher unmet need. This disparity was influenced by factors such as household size, husbands’ attitudes toward family planning, community acceptance, age at first sexual intercourse, and women's age. To address this, targeted interventions should focus on older women, those from larger households, and rural populations, where the unmet need is most pronounced. It is crucial to engage husbands or male partners in reproductive health programs, emphasizing their role in supporting contraceptive use. In addition, comprehensive health education campaigns in rural communities are necessary to counteract myths and misconceptions surrounding contraception.

In line with these findings, it is recommended that policymakers prioritize rural areas in family planning programs, with a focus on providing accessible contraceptive services and counseling. Health education initiatives should be designed to challenge cultural misconceptions and increase awareness about the benefits of family planning. Furthermore, involving community leaders and male partners in family planning advocacy could help foster a more supportive environment for women. Expanding family planning services in rural health facilities and ensuring their affordability and availability are essential steps toward closing the urban–rural gap in unmet need for family planning.

## Data Availability

Publicly available datasets were analyzed in this study. These data can be found here: https://www.pmadata.org.
